# Perforation of the Duodenum

**Published:** 1829-01

**Authors:** 


### PERFORATION OF THE DUODENUM.

*Case of Perforation of the Duodenum, with Discharge of the Contents of the Stomach between the seventh and eighth Ribs.*

M. A. W., aged thirty-nine, eight years a widow, residing in Carden street, Worcester, was admitted at the WORCESTER DISPENSARY, under the care of Dr. STREETEN, March 6th, 1828. At the time of admission, she complained of great pain in the epigastrium and right hypochondrium, aggravated by pressure; the pulse was quick; the bowels irregular, and the stools dark coloured; the tongue furred; she had cough, with muco-purulent expectoration, and hurried breathing; there was a hard painful tumor under the right scapula; the catamenia were irregular, and she was much emaciated.

She is reported to have enjoyed pretty good health till about four years ago, when she was attacked with severe pain in the right side, shooting through to the shoulder; for which she was bled, and had leeches to the side, and took purgative medicines, with partial relief.

In March last, she again became a patient of the dispensary, with similar symptoms. A few days before her admission, a small tumor was perceived, immediately below the right scapula, which increased in size, at first gradually, but afterwards more rapidly, till it formed a swelling about the size of an orange. Leeches were applied to the tumor, and afterwards a poultice. In the course of a few days it broke, and discharged a large quantity of purulent matter, occasionally bloody and fetid. The strength was supported by nourishing diet and port wine, while anodynes were given to procure sleep and allay pain. She took also the sulphate of quinine. Under this treatment an evident improvement took place, and she appeared to gain strength; the acute pain was relieved, but the side, she said, felt as if raw internally. The wound was dressed with simple cerate and bandages, and on the 10th of June the discharge had nearly ceased.

On the 16th of June, a small painful swelling appeared beneath the skin, situated between the seventh and eighth ribs, about an inch and a half anterior to the angle of the ribs. This gradually increased in size till the evening of the 26th. The skin then gave way, and a large quantity of very offensive, dark coloured, bloody matter was discharged. Matter and coagulated blood, of a very fetid smell, continued to come away at intervals till the 1st of July; since which period the discharge has been in greater quantity, and, it is reported, of the same nature as the food which she has taken.

On the 4th of July, she had considerable pain in the right side, and soreness between the two orifices. The appetite was pretty good; the tongue clean and moist, and the bowels regular. She had at times alternations of heat and chilliness, with perspirations, especially during sleep. The pulse was 120, and feeble. She was exceedingly emaciated, and appeared very weak; the cough, (with which, and the expectoration of muco-purulent matter occasionally tinged with blood, she had been affected from the first commencement of her complaints,) was very troublesome.

On the following morning, a cup of coffee was given her, and almost immediately after a quantity of fluid came from the orifice, below the mamma. This was caught in a clean cup: it was clear, of a light brown colour, and had a decided smell of coffee. There was no uneasiness whatever in the left hypochondrium, but she said she felt occasionally as if the drink which she took went towards the back, and up to the right axilla. None has been observed to escape from the wound below the scapula.

On the 26th, Dr. Hastings saw her with Dr. Streeten. The emaciation and debility were then extreme; the pulse was quick and feeble; the appetite had failed; the bowels were rather costive; the cough was exceedingly troublesome. She took a little water at the visit, which almost immediately came out at the wound: it had a sour smell. Some milk-and-water was then given her: this passed through the external orifice five minutes after it had been taken, perfectly curdled. The abdominal muscles were thrown into action previous to the passage of the milk. She died in the course of the night.

*Sectio cadaveris.*—Body reduced to the extreme of emaciation.

Between the seventh and eighth ribs, about an inch and a half from the angle, were two openings; the larger about an inch in length, the smaller large enough to admit a horsebean. The wound looked dark and sloughy. The lower edge of the seventh, and the upper edge of the eighth ribs, were carious for about an inch in the site of the wound.

Abdomen: The left lobe of the liver was greatly enlarged, and of a pale colour, extending across the epigastrium into the left hypochondrium, and displacing the stomach. The right lobe was of a globular shape, about the size of a large heart; its inferior edge was tumid and rounded. It was closely applied to the left lobe; and, at the superior and anterior part, firmly connected with the cartilages of the seventh and eighth ribs, by a strong fibro-cartilaginous structure, in some places upwards of a quarter of an inch in thickness.

The stomach was displaced by the enlarged liver, the larger curvature being situated a little below the umbilicus, and the smaller curvature being applied to, and lying under, the thin convex edge of the left lobe. The cardiac and pyloric portions were elongated and narrowed by the stretching arising from the displacement and depression of the central parts. The pyloric portion of the stomach ascended from the great curvature below the umbilicus, merging beneath the edge of the left lobe of the liver, and then passing between the right and left lobes, by which it was strongly compressed, was attached at and around the pylorus, upon the upper or convex surface of the liver, and in close and intimate connexion with the cartilages of the eighth and ninth ribs. Immediately beyond the pyloric orifice, the duodenum curved downwards from this point of attachment, and passed between the right and left lobes of the liver, through the same channel, and behind the ascending or pyloric portion of the stomach. Beyond this point the duodenum was much contracted, and contained a small quantity of yellow mucus. Exactly at the point where the curve of the duodenum occurred, there was an opening in its coats, which was about two inches and a half from the external orifice, and communicated with it by a channel running obliquely upwards and outwards. This channel proceeded along the inner surface of the cartilages of the eighth and ninth ribs, crossing the intercostal space, and was then continued along the body of the seventh rib, where it terminated in a deep groove in the lower edge of this rib, just before it unites with its cartilage. The channel was formed anteriorly, by a superficial excavation, or rather smooth depression in the thickened cellular structure lining the cartilages of the ribs; its posterior surface was formed by the tough fibro-cartilaginous substance which connected the liver with the inner surface of the ribs.

The mucous membrane of the stomach was perfectly healthy.

Below the stomach was situated the arch of the colon, with the omentum lying across the small intestines, midway between the umbilicus and the pubes.

Thorax: There were strong adhesions between the pleura pulmonalis and costalis, on the right side of the chest. Between the seventh and eighth ribs, immediately below the right scapula, was a communication between the cavity of the thorax and the exterior of the body; and the eighth rib, at this part, was found to be carious. This was the site of the wound in the back, mentioned in the account of the case. There was no appearance of disease on the surface of the pleura pulmonalis, corresponding with this opening. The right lung, at its inferior part, was resting upon, and inseparably connected with, the superior edge of the right lobe of the liver, through the medium of fibro-cartilaginous substance; all traces of the diaphragm in this part being lost. Both lungs were extensively diseased, containing small, firm, white tubercles; some of them advancing to the suppurative stage.

The head was not examined.

It appears, from the above statement, that there must have been an original malformation in the subject of this case; and that the unusual position of the pylorus and upper part of the duodenum, upon the superior or convex surface of the liver, was intimately connected with its extraordinary termination. The repeated attacks of iuflammation of the liver appear to have induced enlargement, with deposition in the parenchymatous structure of that organ; and, subsequently, compression and constriction of the upper part of the duodenum, in consequence of its unusual position between the two lobes. Hence a difficulty arose in the transmission of the alimentary mass from the stomach into the portions of the intestinal canal situated below the constriction. The natural action of the stomach appears to have transmitted the food through the pylorus, and thus to have overcome the compression existing upon the pyloric portion of the stomach. But when the food had passed through the pyloric orifice, the vis a tergo could no longer have acted in a favorable direction, but must have been exerted upon that portion of the duodenum at which the curvature took place; and the passage downwards being impeded by the partial compression arising from its situation between the right and left lobes of the liver, ulcerative absorption occurred exactly at the spot where pressure from behind was most felt, that is, at the point of the curvature. The same causes continuing in operation, produced the gradual formation of the channel in the thickened cellular membrane between the cartilages of the ribs and the liver, till, approaching the intercostal space, it terminated externally, producing caries of the ribs, and ulcerating through the integuments.

